# Integrated Analyses of the Expression and Prognostic Value of EPHB6 in Cervical Cancer and Its Correlation with Immune Infiltrates

**DOI:** 10.1155/2023/2258906

**Published:** 2023-04-17

**Authors:** Siyang Xiang, Mei Wei, Limei Zhao, Anping Lin, Zhengai Xiong

**Affiliations:** ^1^Department of Obstetrics and Gynecology, Chongqing Medical University, Chongqing, China; ^2^Department of Gynaecology, The First People's Hospital of Chongqing Liangjiang New District, Chongqing, China

## Abstract

Among women, cervical cancer (CC) ranks as the third most frequent form of carcinoma and the fourth greatest cancer-related cause of deaths. There is increasing evidence that points to the dysregulation of EPH receptor B6 (EPHB6) in various cancers. On the other hand, neither the expression nor the function of EPHB6 in CC has been researched. In the first part of this investigation, we analyzed the data from the TCGA and discovered that the level of EPHB6 was much lower in CC tissues than in normal cervical tissues. ROC assays revealed that high EPHB6 expression had an AUC value of 0.835 for CC. The survival study revealed that both the overall and disease-specific survivals in this condition were considerably lower among patients who had a low EPHB6 level compared to those who had a high EPHB6 level. It is important to note that the multivariate COX regression analysis indicated that the expression of EPHB6 was an independent predictive factor. In addition to this, the C-indexes and calibration plots of a nomogram derived from multivariate assays revealed an accurate prediction performance among patients with CC. Immune infiltration analysis indicated that the expression of EPHB6 was positively associated with the levels of Tcm, TReg, B cells, T cells, iDC, T helper cells, cytotoxic cells, and DC, while negatively associated with NK CD56bright cells and neutrophils. In summary, the downregulation of EPHB6 was strongly linked to a more aggressive clinical development of CC, suggesting its potential utility as a diagnostic and therapeutic target in CC.

## 1. Introduction

Cervical cancer (CC) is one of the most common gynecological malignant tumors worldwide, which has become a prominent public health issue [1]. It is observed that the incidence of CC ranks second among gynecological malignant tumors, but its mortality rate ranks first among female malignant tumors in the genital tract [2]. As a result, CC has evolved into a condition that poses a risk to the wellbeing of women [3]. The average age at which CC develops is getting younger, which poses a significant risk to women's health and even life, particularly in less-developed nations [4, 5]. Adenocarcinoma, squamous cell carcinoma, and adenosquamous carcinoma are three subtypes of CC that may be identified histologically, which are results of the heterogeneity that characterizes CC [6, 7]. Chemotherapy, radiation, and surgery are the primary treatment options available to people who have colon cancer at the present time [8]. However, the prognosis of patients continues to be dismal as a result of the spread of metastases and the resistance of the cancer to chemotherapy [9, 10]. Oncotherapy is still faced with significant obstacles despite the progress that has been made in the field of health care. These obstacles include a delayed diagnosis, recurrence or metastasis, and cancer-associated mutations [11, 12]. As a result, the investigation of sensitive biomarkers is of utmost importance.

In the human genome, the erythropoietin-producing hepatocytes, Ephs, or receptors make up the biggest family of tyrosine kinase receptors. EphA1-A10 and EphB1-B6 are the names of the two sets of members that make up the Eph receptor subfamily at this point [13]. There are a total of 16 members making up this subfamily. During embryonic development, Eph receptors play a mediating role in the processes of cell compartmentalization and directional cell migration [14]. Ephs are thought to play crucial roles in the invasiveness of cancers due to their ability to govern cell adhesion and migration [15]. Despite the fact that the functions of various EphB receptors in cancers appear to be in conflict with one another, the Eph receptors have been linked to a number of other malignancies. EPHB6 is considered to be a kinase-dead receptor tyrosine kinase due to the fact that its kinase domain is catalytically inactive, which exhibits many changes in amino acids that are normally conserved [16]. The protein EPHB6 is deleted in more aggressive breast cancers, melanomas, and neuroblastomas [17–19]. It has been demonstrated in the past that the epigenetic silencing of the EPHB6 gene caused by promoter methylation is connected to the downregulation of the EPHB6 gene. Additionally, reports on the predictive significance of EPHB6 in tongue squamous cell carcinoma have also been made [20]. However, very little information is available for the expression and function of EPHB6 in CC.

Because it is intimately connected to the investigation of tumor etiology and the responsiveness of immunotherapy, the interaction between tumor microenvironment (TME) and tumors has become an essential element of studies on tumor biology in recent years [21]. The efficacy of immunotherapy in the treatment of several forms of cancer sheds information on the critical function of TME [22]. However, as CC is a heterogeneous illness, it poses a significant obstacle to the development of customized treatments due to the wide variety of phenotypes it exhibits and its poor outlook. Through complex, two-way, and dynamical interactions between tumors and the stroma, CC tumor cells gain a heightened capacity to penetrate and spread into surrounding tissues, according to a growing body of research [23, 24]. In recent years, bioinformatics assays have played a highly significant role in improving both our understanding of various diseases and our abilities to treat them. One of these diseases is cancer. The expression level of certain markers may be used as a reflection of the invasion of particular cell types in tumor tissues by employing algorithms such as ssGSEA [25, 26]. It is possible to assess the significance of the correlations between the infiltration levels of various cell types and the survival rates of patients by analyzing the follow-up information of numerous cohorts in their entirety. As a result, we wanted to investigate the expression of EPHB6 in patients with CC and its predictive relevance. In addition, we investigated whether or not there was a connection between the expression of EPHB6 and TME.

## 2. Materials and Methods

### 2.1. TCGA Data Acquisition

The TCGA database was accessed to get the survival data of patients with CC, together with the RNA transcriptome data presented in the format of FPKM. The TCGA publishing standards were adhered to throughout the entirety of the analysis process. A total of 306 CC specimens and 3 nontumor specimens were enlisted for later studies when duplicate samples from the same individuals were determined to be disregarded. The studies on CC of the TCGA were consulted to get fundamental clinical information pertaining to patients with CC. Following the application of the aforementioned filters, a total of 306 individuals diagnosed with CC in the TCGA dataset were included in this analysis.

### 2.2. Differential Expression of EPHB6 in CC Tissues in the TCGA Database

In order to calculate the differential expression of EPHB6, boxplots and scatter plots were produced using the illness state as a variable. The disease state was either tumorigenic or normal. How well EPHB6 performed as a diagnostic tool was determined through the use of receiver operating characteristic (ROC) curves. It was determined that a statistical ranking of EPHB6 expression that was either higher or lower than the median value was designated as EPHB6-high or EPHB6-low, respectively.

### 2.3. Clinical Statistical Analysis on Prognosis, Model Construction, and Evaluation

The Wilcoxon signed-rank sum test and logistic regression were utilized to delve into the nature of the connection existing between clinical pathologic characteristics and EPHB6. Using Cox regression and the Kaplan–Meier technique, patients with TCGA were analyzed to determine the clinicopathological features that were linked with 10-year overall survival (OS) and disease-specific survival (DSS). In order to evaluate the impact of EPHB6 expression on survivals in conjunction with other clinical parameters, a multivariate Cox regression model was utilized (stage, myometrial invasion, lymph node status, distant metastasis status, histological grade, and subtype). The median value of EPHB6 expression was used to establish the value that served as the cut-off point. In every experiment, a *p* value of less than 0.05 was deemed to indicate statistical significance. Using the Kaplan–Meier technique and a two-sided log-rank test, we were able to determine the difference between the 10-year OS and DSS of groups with a high and low level of EPHB6.

The independent prognostic indicators acquired from a multivariate analysis were utilized to build nomograms, through which the expected survival probability for one 1, 3, and 5 years was individualized. These nomograms were established on the basis of Cox regression models. The RMS software was decidedly used to create nomograms containing important clinical features and calibration plots. The calibration curves were visually evaluated by mapping the nomogram-predicted probability against the occurrences observed; the 45° line represented the best predictive value among all the lines in the assessment. To evaluate the accuracy of discrimination based on the nomogram, a concordance index, abbreviated as C-index, was utilized, whose value was determined using the bootstrap method with a total of 1,000 resamples. The C-index was utilized to make a comparison between the prediction accuracy of the nomogram and that of individual prognostic parameters. In this particular research endeavor, all statistical tests were conducted using two different sets of data, and the threshold of statistical significance was established as 0.05.

### 2.4. Immune Infiltration Analysis

We calculated the infiltration extent of 28 immune cell types using the single-sample gene set enrichment analysis (ssGSEA) method included in the GSVA R package [27]. Our calculations were based on the expression levels of genes included in 28 published gene sets that were associated with immune cells.

### 2.5. Statistical Analysis

All statistical analyses were conducted with R Studio (4.0.2, Boston, USA). All hypothetical tests were two-sided, and a *p* value < 0.05 was considered significant.

## 3. Results

### 3.1. EPHB6 is Downregulated in Human CC Specimens

**Figure 1:**
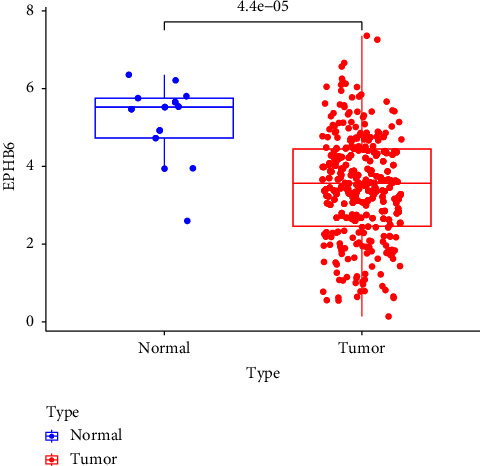
The expression of EPHB6 was analyzed based on TCGA datasets for both CC and non-tumor samples.

In order to evaluate the relevance of the expression of EPHB6 in CC, we began by examining its expression in patients with CC based on TCGA datasets. This allowed us to analyze both the expression of EPHB6 and its significance in CC. The expression of EPHB6 was dramatically downregulated in CC tissues compared to normal cervical tissues, as is illustrated in Figure 1. This difference was statistically significant (*p* < 0.01).

### 3.2. The Diagnostic Significance of EPHB6 Expression in CC

**Figure 2:**
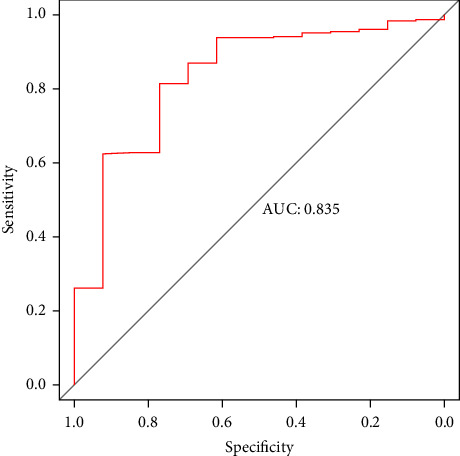
Diagnostic utility of EPHB6 expression in screening CC and non-tumor tissues, as is measured by receiver operating characteristic curves.

Previous research has demonstrated that a number of functional genes have diagnostic significance for patients with CC. After that, we carried out ROC tests, which demonstrated that a high EPHB6 expression possessed an AUC value of 0.835 for CC (Figure 2).

### 3.3. Association of EPHB6 with the Clinicopathological Parameters of CC

Further investigations into the relationships between EPHB6 and the clinicopathological factors of CC were carried out so that we could examine the clinical significance of EPHB6 in CC. According to what is presented in Table 1, our team did not identify a discernible distinction between the expression of EPHB6 and a number of clinical variables, including age and clinical stage.

### 3.4. Prognostic Values of EPHB6 Expression in Patients with CC

**Figure 3:**
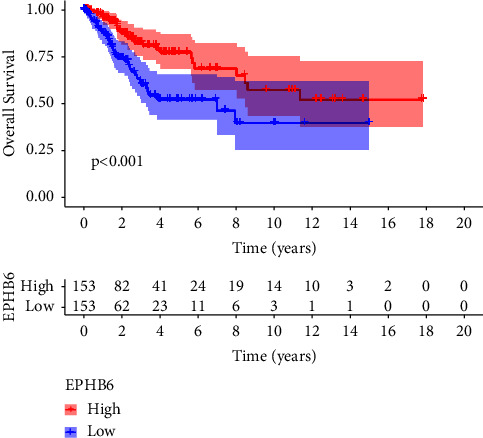
Overall curves of two groups defined by a low and high expression of EPHB6 in patients with CC.

**Figure 4:**
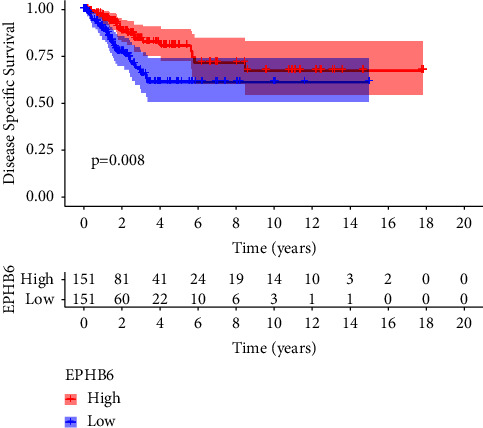
Disease-specific survivals in the two groups defined by a low and high expression of EPHB6 in patients with CC.

The Kaplan–Meier survival curves indicated that the overall survival (Figure 3, *p* = 0.001) and illness-specific survival (Figure 4, *p* = 0.008) of patients with a low EPHB6 level were considerably lower than those of patients with a high EPHB6 level. An investigation using univariate Cox regression revealed that clinical stages and EPHB6 expression had a significant impact on both the overall survival rate and the survival rate specific to the illness (Tables 2 and 3). Additionally, a multivariate COX regression analysis demonstrated that the expression of EPHB6 was an independent predictive predictor for both overall and disease-specific survivals (Tables 2 and 3).

### 3.5. The Construction and Validation of a Nomogram Based on EPHB6

**Figure 5:**
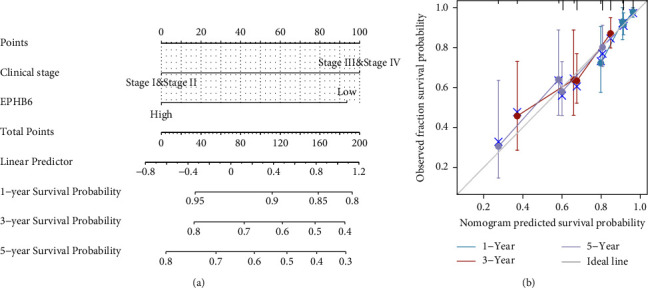
A quantitative approach that can be used to forecast the likelihood of a cancerous patient's overall survival after 1, 3 and 5 years. (a) A nomogram used to estimate the likelihood of 1-year, 3-year and 5-year overall survival of patients with cancers. (b) Plots of calibration based on the nomogram used to forecast the chance of overall survival after 1, 3 and 5 years.

In order to give a quantitative method for predicting the outcome of patients with GC, a nomogram was constructed using EPHB6 in conjunction with other clinical risk indicators that are independent of one another (Figure 5(a)). A point scale was utilized in the construction of a nomogram based on a multivariate Cox analysis. Each variable was given a certain number of points depending on the scale. The total number of points given to each variable was recalculated to fall within the range of 1 to 100. The number of points earned across all the factors was then used as the basis for the final score. A vertical line was drawn immediately downwards from the total point axis to the outcome axis, where the chance of survivals was calculated for patients with CC 1, 3, and 5 years after their diagnosis (Figure 5(a)). We also performed an analysis on the ability of the nomogram to accurately forecast the future, and the findings showed that the C-index of the model was greater than 0.7, which indicated that the ability of the nomogram to accurately predict the future was only moderate. The bias-corrected line in the calibration plot was utilized to be close to the ideal curve, which was the line at 45°, showing that the prediction and the observation were in close agreement with one another (Figure 5(b)). These findings revealed that the nomogram was a more accurate model than individual prognostic indicators for predicting the short- or long-term survival of patients who had CC.

### 3.6. Comparison of Immune Infiltration

**Figure 6:**
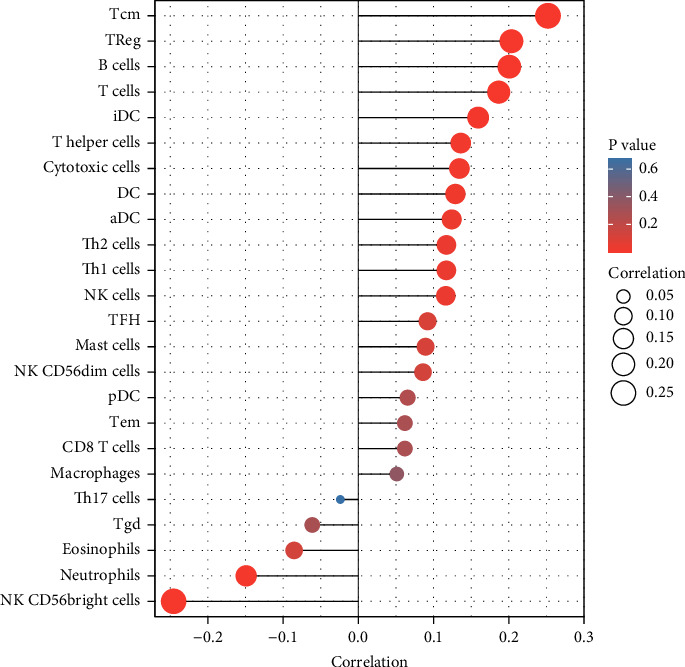
Relationships between EPHB6 and infiltrating immune cells in CC.

We carried out ssGSEA tests so that we could investigate the relationship between the level of EPHB6 and the immune microenvironment. The expression of EPHB6 was shown to be favorably linked with the levels of TCM, TReg, B cells, T cells, iDC, T helper cells, cytotoxic cells, and DC, as is shown in Figure 6. On the other hand, it was found to be negatively associated with NK CD56bright cells and neutrophils. Based on our findings, it seems likely that EPHB6 is involved in the intricate workings of the immunological microenvironment.

## 4. Discussion

In recent years, the technology of microarrays has been used in conjunction with integrated bioinformatic analyses to find new genes associated with a variety of disorders [28, 29]. These genes have the potential to operate as biological markers for diagnosis and prognosis. For instance, Yang et al. reported that the expression level of PAMR1 in cervical cancer tissues was lower than that in normal cervix tissues, which was negatively associated with clinicopathologic characteristics. This is the case for all cervical cancer tissues. A positive prognosis was also predicted for individuals with CC who had a high expression level of PAMR1. The results of the CCK8, transwell, and wound-healing experiments revealed that CC cells were allowed to proliferate, migrate, and invade into surrounding tissues more easily through inhibiting PAMR1 [30]. He et al. showed that the expression of MYO10 was shown to be higher in cancerous cervical tissues and cells compared to normal controls. Furthermore, studies on survivals revealed that patients with a high MYO10 expression had a worse chance to survive the disease overall. In addition, by rewiring the PI3K/Akt signaling pathway of CC caused by the knockdown or overexpression of MYO10, the capability of cervical cells in terms of proliferation, invasion, and migration was dramatically hindered or improved [31]. These findings showed that some tumor-related genes might have the potential to be employed as new biomarkers for the diagnosis and prognosis of individuals suffering from CC.

In the past, a number of studies have revealed the deregulation of EPHB6 in a variety of malignancies, including breast cancers, colorectal cancers and pediatric T-cell acute lymphoblastic leukemia, among others [32–34]. However, whether EPHB6 was operating in an aberrant manner in CC has not been determined. After doing an analysis of TCGA and GTEx data, the first thing that we did in this study was to report that the expression of EPHB6 was significantly lower in CC specimens compared with nontumor samples. According to our findings, EPHB6 might act as a positive regulator in the evolution of CC. The findings of the ROC tests verified the diagnostic utility of EPHB6 expression in screening CC specimens from nontumor ones, which highlighted its potential as a diagnostic biomarker for CC. In addition, a survival study showed that a decreased expression of EPHB6 was connected to a bad outcome of individuals who had CC. It is important to note that the multivariate Cox regression analysis indicated that the expression of EPHB6 was an independent predictive factor for both overall and illness-specific survivals. After that, an exhaustive review was carried out on a nomogram, in which EPHB6 was combined with other significant clinical patterns (clinical stage and EPHB6 specifically), so as to produce a more accurate diagnosis. According to the calibration plot, there was a satisfactory concordance between the observed values and those expected for 1, 3, and 5 years of OS. Our approach was built on a complimentary perspective for each different tumor, which supplied an individualized score for each individual patient. As a consequence of this, our nomogram has the potential to become a very helpful new prognostic tool in the near future.

TME is a multilayered intricate system created when cancer cells interact with the stromal and immune cells in their surroundings [35], which are involved throughout the whole process, from the beginning of the tumor growth to its response to treatment. Patients with colorectal cancers have a highly-immunosuppressive TME, which is one of the primary factors that contributes to their immunotherapy resistance in CC [36, 37]. The accumulation of lactate as a byproduct of aerobic glycolysis results in the formation of an acidic environment that makes tumor penetration easier, which plays a crucial role in the formation of an immunosuppressive TME. The primary components of this immunosuppressive TME are known as tumor-associated macrophages (TAMs), regulatory T cells (Tregs), and myeloid-derived suppressor cells (MDSCs), respectively. It has been shown that these cells can enhance systemic T cell failure, which avoids immune detection and the spread of CC. The results of an examination of immune infiltration indicated that the expression of EPHB6 was favorably linked with the level of TCM, TReg, B cells, T cells, iDC, T helper cells, cytotoxic cells, and DC, but was negatively associated with NK CD56bright cells and neutrophils. As a result, the relationship that exists between EPHB6 and the immune cells may be partially responsible for the anti-cancer impact that it has.

Nonetheless, there are certain limitations. Because the tests took place in different labs, first of all, there was a lack of standardized treatments and a dearth of clinical data in public databases. Secondly, research into the molecular pathways through which EPHB6 might contribute to carcinogenesis was lacking. Future wet lab work is planned to investigate the potential role of EPHB6 in CC signaling pathways.

## 5. Conclusion

Together, our findings suggest that EPHB6 may improve our ability to predict the outcomes of people with CC, encourage the creation of cutting-edge immune-based treatments, and maximize clinical effectiveness. Overall, the possible impact and mechanism of EPHB6 in CC need more investigations.

## Figures and Tables

**Table 1:** Correlation of clinicopathological features of CC with the expression level of EPHB6.

Characteristic	Low expression of EPHB6	High expression of EPHB6	*p*
*n*	153	153	
Age, *n* (%)			0.127
≤50	101 (33%)	87 (28.4%)	
>50	52 (17%)	66 (21.6%)	
Clinical stage, *n* (%)			0.449
Stage I	85 (28.4%)	77 (25.8%)	
Stage II	29 (9.7%)	40 (13.4%)	
Stage III	25 (8.4%)	21 (7%)	
Stage IV	12 (4%)	10 (3.3%)	
Histologic grade, *n* (%)			1.000
G1	9 (3.3%)	10 (3.6%)	
G2	67 (24.5%)	68 (24.8%)	
G3	60 (21.9%)	59 (21.5%)	
G4	0 (0%)	1 (0.4%)	
Age, median (IQR)	45 (37, 55)	47 (40, 59)	0.071

**Table 2:** Univariate and multivariate analyses on the overall survival based on the cox regression model.

Characteristics	Total (*N*)	Univariate analysis		Multivariate analysis	
		Hazard ratio (95% CI)	*p* value	Hazard ratio (95% CI)	*p* value
Age	306				
≤50	188	Reference			
>50	118	1.289 (0.810–2.050)	0.284		
Clinical stage	299				
Stage I and Stage II	231	Reference			
Stage III and Stage IV	68	2.369 (1.457–3.854)	**<0.001**	2.529 (1.551–4.123)	**<0.001**
Histologic grade	274				
G1 and G2	154	Reference			
G3 and G4	120	0.866 (0.514–1.459)	0.589		
EPHB6	306				
Low	153	Reference			
High	153	0.439 (0.272–0.709)	**<0.001**	0.420 (0.259–0.680)	**<0.001**

The bold values mean statistically significant.

**Table 3:** Univariate and multivariate analyses on disease-specific survivals based on the cox regression model.

Characteristics	Total (*N*)	Univariate analysis		Multivariate analysis	
		Hazard ratio (95% CI)	*p* value	Hazard ratio (95% CI)	*p* value
Age	302				
≤50	186	Reference			
>50	116	1.295 (0.761–2.204)	0.340		
Clinical stage	295				
Stage I and Stage II	227	Reference			
Stage III and Stage IV	68	2.675 (1.550–4.615)	**<0.001**	2.800 (1.620–4.839)	**<0.001**
Histologic grade	271				
G1 and G2	152	Reference			
G3 and G4	119	0.922 (0.514–1.654)	0.785		
EPHB6	302				
Low	149	Reference			
High	153	0.473 (0.275–0.813)	**0.007**	0.454 (0.263–0.783)	**0.005**

The bold values mean statistically significant.

## Data Availability

The original data can be obtained from the corresponding authors upon reasonable requests.

## References

[1] Siegel R. L., Miller K. D., Jemal A. (2019). Cancer statistics, 2019. *CA: A Cancer Journal for Clinicians*.

[2] Waggoner S. E. (2003). Cervical cancer. *The Lancet*.

[3] Kim K., Han H. R. (2016). Potential links between health literacy and cervical cancer screening behaviors: a systematic review. *Psycho-Oncology*.

[4] Menderes G., Black J., Schwab C. L., Santin A. D. (2016). Immunotherapy and targeted therapy for cervical cancer: an update. *Expert Review of Anticancer Therapy*.

[5] Johnson C. A., James D., Marzan A., Armaos M. (2019). Cervical cancer: an overview of pathophysiology and management. *Seminars in Oncology Nursing*.

[6] Weyers S., Garland S. M., Cruickshank M., Kyrgiou M., Arbyn M. (2021). Cervical cancer prevention in transgender men: a review. *BJOG: An International Journal of Obstetrics and Gynaecology*.

[7] Revathidevi S., Murugan A. K., Nakaoka H., Inoue I., Munirajan A. K. (2021). APOBEC: a molecular driver in cervical cancer pathogenesis. *Cancer Letters*.

[8] Buskwofie A., David-West G., Clare C. A. (2020). A review of cervical cancer: incidence and disparities. *Journal of the National Medical Association*.

[9] Burd E. M. (2003). Human papillomavirus and cervical cancer. *Clinical Microbiology Reviews*.

[10] Olusola P., Banerjee H. N., Philley J. V., Dasgupta S. (2019). Human papilloma virus-associated cervical cancer and health disparities. *Cells*.

[11] Mauricio D., Zeybek B., Tymon-Rosario J., Harold J., Santin A. D. (2021). Immunotherapy in cervical cancer. *Current Oncology Reports*.

[12] Gadducci A., Cosio S. (2020). Neoadjuvant chemotherapy in locally advanced cervical cancer: review of the literature and perspectives of clinical research. *Anticancer Research*.

[13] Feduniw S., Warzecha D., Szymusik I., Wielgos M. (2020). Epidemiology, prevention and management of early postpartum hemorrhage - a systematic review. *Ginekologia Polska*.

[14] Liang L. Y., Patel O., Janes P. W., Murphy J. M., Lucet I. S. (2019). Eph receptor signalling: from catalytic to non-catalytic functions. *Oncogene*.

[15] Shiuan E., Chen J. (2016). Eph receptor tyrosine kinases in tumor immunity. *Cancer Research*.

[16] Mason E. O., Goldgur Y., Robev D., Freywald A., Nikolov D. B., Himanen J. P. (2021). Structure of the EphB6 receptor ectodomain. *PLoS One*.

[17] Zangrossi M., Romani P., Chakravarty P. (2021). EphB6 regulates TFEB-lysosomal pathway and survival of disseminated indolent breast cancer cells. *Cancers*.

[18] Hafner C., Bataille F., Meyer S. (2003). Loss of EphB6 expression in metastatic melanoma. *International Journal of Oncology*.

[19] Tang X. X., Evans A. E., Zhao H. (1999). High-level expression of EPHB6, EFNB2, and EFNB3 is associated with low tumor stage and high TrkA expression in human neuroblastomas. *Clinical Cancer Research: An Official Journal of the American Association for Cancer Research*.

[20] Dong Y., Pan J., Ni Y., Huang X., Chen X., Wang J. (2015). High expression of EphB6 protein in tongue squamous cell carcinoma is associated with a poor outcome. *International Journal of Clinical and Experimental Pathology*.

[21] Arneth B. (2019). Tumor microenvironment. *Medicina*.

[22] Jarosz-Biej M., Smolarczyk R., Cichoń T., Kułach N. (2019). Tumor microenvironment as A “game changer” in cancer radiotherapy. *International Journal of Molecular Sciences*.

[23] Butturini E., Carcereri de Prati A., Boriero D., Mariotto S. (2019). Tumor dormancy and interplay with hypoxic tumor microenvironment. *International Journal of Molecular Sciences*.

[24] Lodewijk I., Nunes S. P., Henrique R., Jerónimo C., Dueñas M., Paramio J. M. (2021). Tackling tumor microenvironment through epigenetic tools to improve cancer immunotherapy. *Clinical Epigenetics*.

[25] Le T., Aronow R. A., Kirshtein A., Shahriyari L. (2021). A review of digital cytometry methods: estimating the relative abundance of cell types in a bulk of cells. *Briefings in Bioinformatics*.

[26] Li X., Cheng Y., Cheng Y., Shi H. (2022). Transcriptome analysis reveals the immune infiltration profiles in cervical cancer and identifies KRT23 as an immunotherapeutic target. *Frontiers in Oncology*.

[27] Hänzelmann S., Castelo R., Guinney J. (2013). GSVA: gene set variation analysis for microarray and RNA-seq data. *BMC Bioinformatics*.

[28] Kok V. C., Yu C. C. (2020). Cancer-Derived exosomes: their role in cancer biology and biomarker development. *International Journal of Nanomedicine*.

[29] Pessoa L. S., Heringer M., Ferrer V. P. (2020). ctDNA as a cancer biomarker: a broad overview. *Critical Reviews In Oncology-Hematology*.

[30] Yang R., Ma M., Yu S., Li X., Zhang J., Wu S. (2021). High expression of PAMR1 predicts favorable prognosis and inhibits proliferation, invasion, and migration in cervical cancer. *Frontiers in Oncology*.

[31] He J. H., Chen J. G., Zhang B. (2020). Elevated MYO10 predicts poor prognosis and its deletion hampers proliferation and migration potentials of cells through rewiring PI3K/akt signaling in cervical cancer. *Technology in Cancer Research and Treatment*.

[32] Kandpal R. P. (2010). Tyrosine kinase-deficient EphB6 receptor-dependent alterations in proteomic profiles of invasive breast carcinoma cells as determined by difference gel electrophoresis. *Cancer Genomics and Proteomics*.

[33] Wang J., Zhang Y., Ma J. (2021). Determining the effects of Ephrin Type B Receptor 6 and Type A Receptor 3 on facilitating colorectal epithelial cell malignant transformation. *Neoplasma*.

[34] El Zawily A., McEwen E., Toosi B. (2017). The EphB6 receptor is overexpressed in pediatric T cell acute lymphoblastic leukemia and increases its sensitivity to doxorubicin treatment. *Scientific Reports*.

[35] Bejarano L., Jordāo M. J. C., Joyce J. A. (2021). Therapeutic targeting of the tumor microenvironment. *Cancer Discovery*.

[36] Laplane L., Duluc D., Bikfalvi A., Larmonier N., Pradeu T. (2019). Beyond the tumour microenvironment. *International Journal of Cancer*.

[37] Cao S., Lin C., Li X., Liang Y., Saw P. E. (2021). TME-responsive multistage nanoplatform for siRNA delivery and effective cancer therapy. *International Journal of Nanomedicine*.

